# [*N*,*N*′-Bis(2,6-dichloro­benzyl­idene)propane-1,3-diamine-κ^2^ *N*,*N*′]dibromidozinc

**DOI:** 10.1107/S1600536812027997

**Published:** 2012-06-23

**Authors:** Aliakbar Dehno Khalaji, Gholamhossein Grivani, Mohammad Seyyedi, Karla Fejfarová, Michal Dušek

**Affiliations:** a Department of Chemistry, Faculty of Science, Golestan University, Gorgan, Iran; b School of Chemistry, Damghan University, Damghan 36715-364, Iran; c Institute of Physics ASCR, v.v.i., Na Slovance 2, 182 21 Praha 8, Czech Republic

## Abstract

In the title compound, [ZnBr_2_(C_17_H_14_Cl_4_N_2_)], the Zn^II^ ion is bonded to two bromide ions and two N atoms of the diimine ligand and displays a moderately distorted tetra­hedral coordination geometry. The Schiff base ligand acts as a chelating ligand and coordinates to the Zn^II^ atom *via* two N atoms.

## Related literature

For related structures, see: Khalaj *et al.* (2008 ▶, 2009 ▶); Saleh­zadeh *et al.* (2011 ▶); Khalaji *et al.* (2010 ▶, 2011 ▶, 2012 ▶). For properties and application of complexes of symmetric bidentate Schiff base ligands, see: Komatsu *et al.* (2007 ▶); Montazerozohori *et al.* (2011 ▶). For bond-length data, see: Allen *et al.* (1987 ▶).
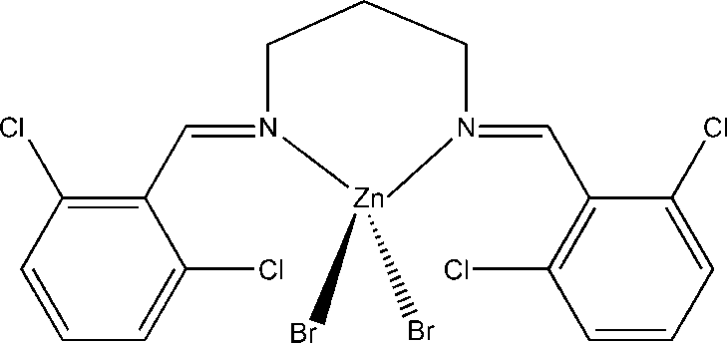


## Experimental

### 

#### Crystal data

[ZnBr_2_(C_17_H_14_Cl_4_N_2_)]
*M* *_r_* = 613.3
Monoclinic, 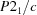

*a* = 17.0433 (3) Å
*b* = 9.3216 (2) Å
*c* = 13.6038 (2) Å
β = 97.313 (2)°
*V* = 2143.67 (7) Å^3^
*Z* = 4
Mo *K*α radiation
μ = 5.38 mm^−1^
*T* = 120 K
0.33 × 0.28 × 0.10 mm

#### Data collection

Agilent Xcalibur diffractometer with an Atlas (Gemini ultra Cu) detector
Absorption correction: multi-scan (*CrysAlis PRO*; Agilent, 2011 ▶) *T*_min_ = 0.5, *T*_max_ = 1
32456 measured reflections
5469 independent reflections
4344 reflections with *I* > 3σ(*I*)
*R*_int_ = 0.032

#### Refinement

*R*[*F* ^2^ > 3σ(*F* ^2^)] = 0.023
*wR*(*F* ^2^) = 0.053
*S* = 1.32
5469 reflections
235 parameters
H-atom parameters constrained
Δρ_max_ = 0.55 e Å^−3^
Δρ_min_ = −0.43 e Å^−3^

### 

Data collection: *CrysAlis PRO* (Agilent, 2011 ▶); cell refinement: *CrysAlis PRO*; data reduction: *CrysAlis PRO*; program(s) used to solve structure: *SIR2002* (Burla *et al.*, 2003 ▶); program(s) used to refine structure: *JANA2006* (Petříček *et al.*, 2006 ▶); molecular graphics: *DIAMOND* (Brandenburg & Putz, 2005 ▶); software used to prepare material for publication: *JANA2006*.

## Supplementary Material

Crystal structure: contains datablock(s) global, I. DOI: 10.1107/S1600536812027997/bt5947sup1.cif

Structure factors: contains datablock(s) I. DOI: 10.1107/S1600536812027997/bt5947Isup2.hkl

Additional supplementary materials:  crystallographic information; 3D view; checkCIF report

## Figures and Tables

**Table 1:** Selected bond lengths (Å)

Zn1—Br1	2.3599 (3)
Zn1—Br2	2.3371 (3)
Zn1—N1	2.0662 (16)
Zn1—N2	2.0628 (16)

**Fig. 1.**
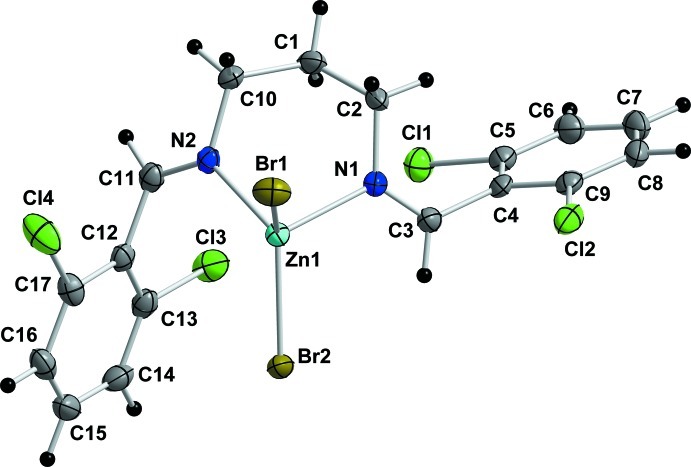
The molecular structure of 1. Displacement ellipsoids are drawn at the 50% probability level.

## supplementary crystallographic information

### Comment

Complexes of symmetric bidentate Schiff base ligands with transition metals have attracted much attention because of their catalytic (Komatsu *et al.*, 2007) and thermal properties (Montazerozohori *et al.*, 2011). There is substantial interest in the coordination chemistry of the zinc(II) ion (Khalaj *et al.*, 2008, 2009; Salehzadeh *et al.*,2011; Khalaji *et al.*,2010, 2011, 2012).

The molecular structure of 1 with the atom-numbering scheme is presented in Fig. 1, and the bond lengths and angles are generally normal (Allen *et al*., 1987). The zinc(II) ion is coordinated by the bidentate Schiff-base ligand and two Br ions. Although a tetrahedral geometry might be expected for a four coordinated zinc(II) centre, the geometry around the zinc(II) ion is distorted by the bite angle N1—Zn1—N2 [90.24 (6)°] of the chelating ligand. On the contrary the Br1—Zn11—Br2 angle has opened up to 120.866 (11)°. The N—Zn—Br angles are also distorted from the tetrahedral values.

### Experimental

To a stirring solution of the (2,6-Cl-ba)_2_en ligand (1 mmol, in 5 ml of chloroform) was added ZnBr_2_ (1 mmol) in 10 ml of methanol and the mixture was stirred for 10 min in air at room temperature and was then left at 273 K for several days without disturbance yielding suitable crystals that subsequently were filtered off and washed with Et_2_O.

### Refinement

All H atoms were positioned geometrically and treated as riding on their parent atoms. The displacement coefficients *U*_iso_(H) were set to 1.2*U*_eq_(C).

### Crystal data

**Table:**

[ZnBr_2_(C_17_H_14_Cl_4_N_2_)]	*F*(000) = 1192
*M**_r_* = 613.3	*D*_x_ = 1.900 Mg m^−^^3^
Monoclinic, *P*2_1_/*c*	Mo *K*α radiation, λ = 0.7107 Å
Hall symbol: -P 2ybc	Cell parameters from 14022 reflections
*a* = 17.0433 (3) Å	θ = 3.0–29.3°
*b* = 9.3216 (2) Å	µ = 5.38 mm^−^^1^
*c* = 13.6038 (2) Å	*T* = 120 K
β = 97.313 (2)°	Block, colourless
*V* = 2143.67 (7) Å^3^	0.33 × 0.28 × 0.10 mm
*Z* = 4	

### Data collection

**Table:**

Agilent Xcalibur diffractometer with an Atlas (Gemini ultra Cu) detector	5469 independent reflections
Radiation source: Enhance (Mo) X-ray Source	4344 reflections with *I* > 3σ(*I*)
Graphite monochromator	*R*_int_ = 0.032
Detector resolution: 10.3784 pixels mm^-1^	θ_max_ = 29.4°, θ_min_ = 3.0°
Rotation method data acquisition using ω scans	*h* = −21→23
Absorption correction: multi-scan (*CrysAlis PRO*; Agilent, 2011)	*k* = −12→12
*T*_min_ = 0.5, *T*_max_ = 1	*l* = −18→17
32456 measured reflections	

### Refinement

**Table:**

Refinement on *F*^2^	56 constraints
*R*[*F*^2^ > 2σ(*F*^2^)] = 0.023	H-atom parameters constrained
*wR*(*F*^2^) = 0.053	Weighting scheme based on measured s.u.'s *w* = 1/(σ^2^(*I*) + 0.0004*I*^2^)
*S* = 1.32	(Δ/σ)_max_ = 0.002
5469 reflections	Δρ_max_ = 0.55 e Å^−^^3^
235 parameters	Δρ_min_ = −0.43 e Å^−^^3^
0 restraints	

### Special details

**Table:**

Experimental. Absorption correction: CrysAlisPro (Agilent Technologies, 2011) Empirical absorption correction using spherical harmonics, implemented in SCALE3 ABSPACK scaling algorithm.
Refinement. The refinement was carried out against all reflections. The conventional *R*-factor is always based on *F*. The goodness of fit as well as the weighted *R*-factor are based on *F* and *F*^2^ for refinement carried out on *F* and *F*^2^, respectively. The threshold expression is used only for calculating *R*-factors *etc*. and it is not relevant to the choice of reflections for refinement.The program used for refinement, Jana2006, uses the weighting scheme based on the experimental expectations, see _refine_ls_weighting_details, that does not force *S* to be one. Therefore the values of *S* are usually larger than the ones from the *SHELX* program.

### Fractional atomic coordinates and isotropic or equivalent isotropic displacement parameters (Å^2^)

**Table:**

	*x*	*y*	*z*	*U*_iso_*/*U*_eq_	
Zn1	0.778715 (13)	−0.01264 (2)	0.739863 (16)	0.01974 (7)	
Br1	0.815993 (14)	−0.06312 (2)	0.909367 (15)	0.03043 (7)	
Br2	0.768458 (12)	0.22386 (2)	0.682623 (15)	0.02510 (7)	
Cl1	0.57897 (3)	−0.18717 (6)	0.46139 (4)	0.03164 (17)	
Cl2	0.49527 (3)	−0.02131 (6)	0.81149 (4)	0.03069 (16)	
Cl3	0.78647 (4)	−0.01110 (6)	0.42235 (4)	0.03640 (18)	
Cl4	1.01713 (4)	−0.02162 (7)	0.73027 (4)	0.0435 (2)	
N1	0.67043 (10)	−0.11134 (16)	0.70285 (12)	0.0204 (5)	
N2	0.83223 (9)	−0.16701 (17)	0.66248 (11)	0.0190 (5)	
C1	0.72353 (12)	−0.3466 (2)	0.66028 (16)	0.0272 (6)	
C2	0.67041 (12)	−0.2659 (2)	0.72376 (16)	0.0273 (6)	
C3	0.60879 (12)	−0.0487 (2)	0.66383 (14)	0.0216 (6)	
C4	0.52967 (11)	−0.1151 (2)	0.63559 (15)	0.0219 (6)	
C5	0.50947 (12)	−0.1818 (2)	0.54428 (15)	0.0252 (6)	
C6	0.43508 (13)	−0.2408 (2)	0.51740 (17)	0.0321 (7)	
C7	0.37960 (13)	−0.2328 (2)	0.58284 (17)	0.0342 (7)	
C8	0.39751 (12)	−0.1666 (2)	0.67387 (17)	0.0306 (7)	
C9	0.47174 (12)	−0.1085 (2)	0.69829 (15)	0.0240 (6)	
C10	0.81108 (12)	−0.3168 (2)	0.68397 (14)	0.0218 (6)	
C11	0.87837 (12)	−0.1473 (2)	0.59844 (14)	0.0232 (6)	
C12	0.90508 (12)	−0.0029 (2)	0.57246 (14)	0.0223 (6)	
C13	0.86796 (12)	0.0692 (2)	0.48978 (15)	0.0248 (6)	
C14	0.89235 (13)	0.2026 (2)	0.46228 (17)	0.0308 (7)	
C15	0.95599 (13)	0.2659 (2)	0.51856 (16)	0.0312 (7)	
C16	0.99483 (13)	0.1988 (2)	0.60108 (16)	0.0300 (7)	
C17	0.96911 (12)	0.0647 (2)	0.62674 (15)	0.0260 (6)	
H1a	0.714222	−0.447812	0.664797	0.0326*	
H1b	0.706896	−0.326754	0.591541	0.0326*	
H2a	0.617401	−0.302232	0.710663	0.0327*	
H2b	0.688664	−0.281934	0.792584	0.0327*	
H3	0.613239	0.052004	0.650974	0.0259*	
H6	0.422365	−0.286607	0.45416	0.0386*	
H7	0.328059	−0.273674	0.564947	0.0411*	
H8	0.358864	−0.161171	0.719285	0.0367*	
H10a	0.827835	−0.337727	0.752531	0.0262*	
H10b	0.840072	−0.381379	0.64704	0.0262*	
H11	0.896906	−0.229113	0.56525	0.0278*	
H14	0.865572	0.250488	0.405122	0.037*	
H15	0.973645	0.35864	0.499922	0.0375*	
H16	1.038872	0.244152	0.639989	0.036*	

### Atomic displacement parameters (Å^2^)

**Table:**

	*U*^11^	*U*^22^	*U*^33^	*U*^12^	*U*^13^	*U*^23^
Zn1	0.01828 (12)	0.01772 (12)	0.02378 (12)	−0.00064 (9)	0.00483 (9)	−0.00078 (9)
Br1	0.04287 (14)	0.02484 (11)	0.02295 (11)	−0.00147 (9)	0.00178 (9)	0.00022 (8)
Br2	0.02661 (12)	0.01722 (10)	0.03323 (12)	0.00196 (8)	0.01065 (9)	0.00062 (8)
Cl1	0.0296 (3)	0.0355 (3)	0.0303 (3)	0.0012 (2)	0.0057 (2)	−0.0026 (2)
Cl2	0.0303 (3)	0.0317 (3)	0.0313 (3)	0.0085 (2)	0.0086 (2)	0.0035 (2)
Cl3	0.0359 (3)	0.0301 (3)	0.0389 (3)	0.0017 (2)	−0.0119 (3)	−0.0036 (2)
Cl4	0.0394 (4)	0.0537 (4)	0.0335 (3)	−0.0191 (3)	−0.0102 (3)	0.0086 (3)
N1	0.0173 (8)	0.0191 (8)	0.0252 (8)	0.0008 (7)	0.0047 (7)	0.0012 (7)
N2	0.0159 (8)	0.0209 (8)	0.0194 (8)	0.0000 (7)	−0.0008 (7)	−0.0022 (7)
C1	0.0230 (11)	0.0162 (10)	0.0405 (12)	−0.0015 (8)	−0.0033 (9)	0.0002 (9)
C2	0.0165 (10)	0.0208 (10)	0.0439 (13)	−0.0020 (8)	0.0016 (9)	0.0114 (9)
C3	0.0220 (11)	0.0187 (10)	0.0251 (10)	0.0001 (8)	0.0067 (9)	−0.0002 (8)
C4	0.0161 (10)	0.0181 (10)	0.0312 (10)	0.0046 (8)	0.0019 (8)	0.0056 (8)
C5	0.0222 (11)	0.0218 (10)	0.0315 (11)	0.0032 (8)	0.0038 (9)	0.0042 (9)
C6	0.0275 (12)	0.0282 (11)	0.0387 (13)	−0.0011 (9)	−0.0034 (10)	0.0014 (10)
C7	0.0188 (11)	0.0308 (12)	0.0508 (15)	−0.0022 (9)	−0.0042 (10)	0.0084 (11)
C8	0.0193 (11)	0.0291 (11)	0.0444 (13)	0.0042 (9)	0.0078 (10)	0.0108 (10)
C9	0.0224 (11)	0.0212 (10)	0.0283 (10)	0.0059 (8)	0.0029 (9)	0.0060 (9)
C10	0.0210 (10)	0.0171 (9)	0.0270 (10)	0.0031 (8)	0.0015 (8)	0.0025 (8)
C11	0.0194 (10)	0.0253 (10)	0.0243 (10)	0.0003 (8)	0.0006 (8)	−0.0043 (9)
C12	0.0207 (10)	0.0235 (10)	0.0238 (10)	0.0017 (8)	0.0075 (8)	−0.0045 (8)
C13	0.0222 (11)	0.0244 (10)	0.0275 (10)	0.0028 (8)	0.0026 (9)	−0.0071 (9)
C14	0.0345 (13)	0.0234 (11)	0.0352 (12)	0.0092 (9)	0.0067 (10)	0.0022 (9)
C15	0.0307 (12)	0.0211 (10)	0.0444 (13)	−0.0022 (9)	0.0147 (11)	−0.0012 (10)
C16	0.0254 (12)	0.0305 (12)	0.0355 (12)	−0.0097 (9)	0.0090 (10)	−0.0074 (10)
C17	0.0230 (11)	0.0325 (11)	0.0228 (10)	−0.0038 (9)	0.0042 (9)	−0.0024 (9)

### Geometric parameters (Å, º)

**Table:**

Zn1—Br1	2.3599 (3)	C6—C7	1.381 (3)
Zn1—Br2	2.3371 (3)	C6—H6	0.96
Zn1—N1	2.0662 (16)	C7—C8	1.383 (3)
Zn1—N2	2.0628 (16)	C7—H7	0.96
N1—C2	1.469 (2)	C8—C9	1.377 (3)
N1—C3	1.259 (2)	C8—H8	0.96
N2—C10	1.481 (2)	C10—H10a	0.96
N2—C11	1.259 (3)	C10—H10b	0.96
C1—C2	1.526 (3)	C11—C12	1.478 (3)
C1—C10	1.512 (3)	C11—H11	0.96
C1—H1a	0.96	C12—C13	1.391 (3)
C1—H1b	0.96	C12—C17	1.388 (3)
C2—H2a	0.96	C13—C14	1.379 (3)
C2—H2b	0.96	C14—C15	1.378 (3)
C3—C4	1.489 (3)	C14—H14	0.96
C3—H3	0.96	C15—C16	1.379 (3)
C4—C5	1.393 (3)	C15—H15	0.96
C4—C9	1.386 (3)	C16—C17	1.384 (3)
C5—C6	1.388 (3)	C16—H16	0.96
			
Br1—Zn1—Br2	120.866 (11)	C5—C6—H6	120.45
Br1—Zn1—N1	105.69 (5)	C7—C6—H6	120.45
Br1—Zn1—N2	106.14 (4)	C6—C7—C8	120.7 (2)
Br2—Zn1—N1	108.19 (4)	C6—C7—H7	119.65
Br2—Zn1—N2	120.47 (4)	C8—C7—H7	119.65
N1—Zn1—N2	90.24 (6)	C7—C8—C9	118.9 (2)
Zn1—N1—C2	114.32 (12)	C7—C8—H8	120.57
Zn1—N1—C3	124.61 (13)	C9—C8—H8	120.57
C2—N1—C3	121.05 (16)	C4—C9—C8	122.59 (19)
Zn1—N2—C10	115.00 (12)	N2—C10—C1	112.92 (15)
Zn1—N2—C11	127.37 (14)	N2—C10—H10a	109.47
C10—N2—C11	117.60 (17)	N2—C10—H10b	109.47
C2—C1—C10	115.44 (16)	C1—C10—H10a	109.47
C2—C1—H1a	109.47	C1—C10—H10b	109.47
C2—C1—H1b	109.47	H10a—C10—H10b	105.79
C10—C1—H1a	109.47	N2—C11—C12	122.46 (18)
C10—C1—H1b	109.47	N2—C11—H11	118.77
H1a—C1—H1b	102.77	C12—C11—H11	118.77
N1—C2—C1	111.04 (17)	C11—C12—C13	120.71 (17)
N1—C2—H2a	109.47	C11—C12—C17	122.07 (17)
N1—C2—H2b	109.47	C13—C12—C17	117.19 (18)
C1—C2—H2a	109.47	C12—C13—C14	122.22 (18)
C1—C2—H2b	109.47	C13—C14—C15	118.60 (19)
H2a—C2—H2b	107.85	C13—C14—H14	120.7
N1—C3—C4	126.59 (18)	C15—C14—H14	120.7
N1—C3—H3	116.71	C14—C15—C16	121.4 (2)
C4—C3—H3	116.71	C14—C15—H15	119.32
C3—C4—C5	121.91 (19)	C16—C15—H15	119.32
C3—C4—C9	121.04 (17)	C15—C16—C17	118.73 (19)
C5—C4—C9	117.01 (18)	C15—C16—H16	120.63
C4—C5—C6	121.7 (2)	C17—C16—H16	120.63
C5—C6—C7	119.1 (2)	C12—C17—C16	121.89 (18)
